# Striosomal ghrelin receptor activity controls reward-cost integration

**DOI:** 10.21203/rs.3.rs-10122988/v1

**Published:** 2026-06-25

**Authors:** Alexis A. Salcido, Neftali F. Reyes, Andrea Y. Macias, Serina A. Batson, Dirk W. Beck, Kenichiro Negishi, Cory N. Heaton, Atanu Giri, Luis D. Davila, Raquel J. Ibáñez Alcalá, Lara I. Rakocevic, Hui-Ling Wang, Eduardo Montañez, Celine N. Orozco, Mia Maestas, Alma C. Moreno-Dominguez, Carlos Granillo, Ian R. Wickersham, Yavin Shaham, Travis M. Moschak, Ki A. Goosens, Alexander Friedman

**Affiliations:** 1. Department of Biological Sciences, University of Texas at El Paso, El Paso, TX, USA; 2. Computational Science Program, University of Texas at El Paso, El Paso, TX, USA; 3. Neurobiology of Relapse Section, National Institute on Drug Abuse Intramural Research Program, Baltimore, MD, USA.; 4. McGovern Institute for Brain Research, Massachusetts Institute of Technology, Cambridge, MA 02139, USA; 5. Department of Psychiatry, Department of Pharmacological Sciences; Icahn School of Medicine at Mount Sinai, New York, NY, United States; 6. Institute for Translational Medicine and Pharmacology, Icahn School of Medicine at Mount Sinai, New York, NY, United States

## Abstract

Growth hormone secretagogue receptors (GHSRs) modulate reward and cost processing separately, but their role in conflict-based decision-making is unclear. We observed dose-dependent effects of the GHSR agonist ibutamoren (IBU) on rat decision-making performance. Moderate, but not low or high doses, increased cost sensitivity selectively in conflict paradigms. We observed dense GHSR expression in dorsomedial striosomes, a region critical for conflict decision-making. Moderate GHSR activation increased neuronal activity in dorsomedial striosomes that project to lateral habenula (LHb) and enhanced inactivation of dopaminergic neurons in the substantia nigra pars compacta (daSNc). Chemogenetic manipulation of dorsomedial striosomes activity confirmed their causal influence on both GHSR-mediated increases in cost-sensitivity and circuit activity in LHb and daSNc. Our results identify a novel circuit mechanism linking endocrine activity with conflict decision-making.

Most of our daily decisions involve weighing potential costs against potential rewards, termed “conflict” decision-making (1). Decision-making is influenced by external factors, like whether an object is rewarding or aversive, as well as internal states mediated by endocrine signaling (2), such as hunger (3). However, the neural circuit mechanisms recruited by internal endocrine signals to regulate decision-making are largely unexplored, especially in the context of conflict decision-making. One internal signal that communicates internal state is the gut hormone acyl-ghrelin (hereafter referred to as “ghrelin”). Ghrelin is the endogenous agonist for the growth hormone secretagogue receptor (GHSR; the ghrelin receptor; for abbreviations see table S1) (4), and stimulating GHSRs with ghrelin or ghrelin mimetics (5) can increase or decrease appetite and feeding behaviors (6–8). Ghrelin and elevated GHSR activity have been implicated in non-food reward processing and cost perception (9–14). Further, ghrelin levels are altered in several disorders, including major depressive disorder (15), post-traumatic stress disorder (16), and substance use disorder (17). Identifying underlying hormonal mechanisms that alter conflict decision-making may facilitate future treatments.

The striosomes, a compartment within the striatum, are uniquely important for integrating rewards and costs (18, 19). Striosomes receive selective inputs from cortical regions critical for decision-making (18–21). Striosomes, but not surrounding matrix, send signals directly to the dopaminergic neurons of the substantia nigra pars compacta (daSNc) (22–28). Striosomes also innervate daSNc indirectly through the globus pallidus internal (GPi)→lateral habenula (LHb)→rostral medial tegmental nucleus (RMTg)→daSNc circuit (29, 30). Our computational work suggests that striosomes leverage these two pathways to modulate dopamine release and influence decision-making when conflict is present (e.g., when a potential reward involves experiencing an undesirable cost) (19). Our model posits that during conflict decision-making, the striosome-centric circuit sets a state used for selecting which cortical inputs (including, but not limited to, reward and cost signals) to prioritize (19). Collectively, current evidence and theoretical models place striosomes as an ideal target for GHSR activity to influence cost-benefit conflict decision-making, but the expression of GHSR in striosomes has yet to be evaluated.

To uncover how GHSR activity affects decision-making, we manipulated GHSR activity in rats performing a series of non-conflict (offering a single aversive or rewarding stimulus) or conflict (offering a combination of reward with an aversive cost) decision-making tasks. We found that stimulating GHSRs promoted cost sensitivity only during conflict decisions. We then examined expression of the immediate-early gene cFos across 50 brain regions after GHSR stimulation and found the striosomes and LHb had the greatest increase in neuronal activity. We also examined regions within the striosomal circuit for neural inactivity (31) following GHSR stimulation by measuring phosphorylated pyruvate dehydrogenase (pPDH) expression. We found that GHSR agonists increased inactivity levels of daSNc. Quantitative analysis of GHSR expression levels within the striatum revealed selectively dense expression in striosomes of the dorsomedial striatum (DMS) relative to surrounding matrix. We next used a striosome-specific *Oprm1-Cre* knock-in rat line to chemogenetically manipulate striosomes (32). We found that striosomes are necessary and sufficient for GHSR-mediated modulation of conflict decision-making. Consequently, we propose a computational model wherein GHSR activity within the DMS striosomes narrows the processing of context-relevant information and, when elevated, enhances sensitivity to cost-related signals.

## Results

### GHSR activation dose-dependently affects conflict decision-making

**Fig. 1:**
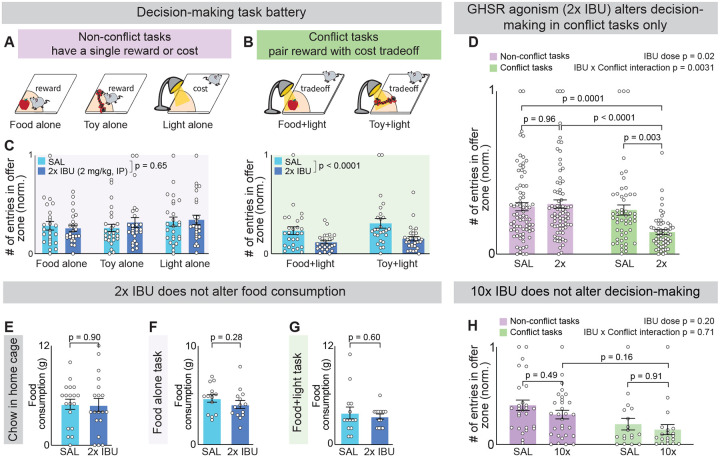
Administering a 2x dose of IBU selectively affects conflict decision-making. **(A-C)** Schematic of **(A)** non-conflict tasks containing a single reward (Food alone, Toy alone) or cost (Light alone), and **(B)** conflict tasks combining reward and cost (Food+light, Toy+light). **(C)** 2x IBU did not alter offer-zone detections across non-conflict tasks (left, rats=25–26/group; two-way ANOVA, main effect of IBU, p=0.65), but significantly reduced detections across conflict tasks (right, rats=26–30/group; p<0.0001). **(D)** Comparison of normalized non-conflict (rats=77) and conflict (rats=52–56) tasks revealed a significant IBU x task interaction (two-way ANOVA, p=0.0031), indicating that the effects of 2x IBU depend on conflict level. **(E–G)** 2x IBU did not alter food consumption in the home cage (**E**, rats=19, paired t-test, p=0.90) or during behavioral testing (**F**, Food alone, rats=14, p=0.28; **G**, Food+light, rats=14, p=0.60). **(H)** 10x IBU did not affect performance across any task (rats=9–10/group; two-way ANOVA, main effect of IBU, p=0.20) and showed no interaction with task conflict (p=0.71). Data were min-max normalized to enable comparisons across tasks with different reward and cost variables. Non-normalized analyses are provided in **fig. S1–2**. Mean ± SEM. For additional statistical analyses see table S3.

To explore how GHSR impacts the integration of reward and cost during decision-making, we designed a battery of approach-avoid decision-making tasks that presented rats with rewards and costs independently or simultaneously (Fig. 1A–B; see Materials and Methods Section 4). One segment of our task battery presented rats with a single rewarding or aversive stimulus within an arena, and the number of times they chose to enter the offer region containing the stimulus was determined. In subsequent conflict tasks, we paired one of the rewarding stimuli with an aversive light, which required the rats to weigh reward against cost when deciding to approach or avoid the joint offer (Food+light or Toy+light).

To manipulate GHSR activity during these tasks, we used ibutamoren mesylate (IBU; see table S2 for reagents), a water-soluble, brain penetrant GHSR agonist, and compared this to saline (SAL). IBU has a half-life of ~6 hours (5, 33); therefore, a single intraperitoneal (IP) injection increases brain GHSR activity for the duration of our behavioral tasks. We tested both a “2x” (2 mg/kg b.w.) dose and a “10x” (10 mg/kg b.w.) dose of IBU administered 120 minutes prior to task performance (Materials and Methods section 4).

In single-stimulus control conditions (Food, Toy, or Light alone) in which we confirmed that the offer zone was approached more often when it contained a reward than when it contained nothing or the aversive light, (p=0.009; **fig. S1A**; for detailed statistical analysis see table S3), 2x IBU did not alter the number of entries into the offer zone compared to SAL (p=0.65; Fig. 1C, min-max normalization was applied to compare across task types; **fig. S1B-D** for non-normalized counts). In contrast, during conflict tasks where the food or toy was paired with an aversive light, 2x IBU reduced the number of times the rat entered the Food+light or Toy+light zone (p<0.0001; **fig. S1E-G**), suggesting either a GSHR-mediated increased sensitivity to cost or decreased sensitivity to reward selectively in the conflict task (IBU-conflict interaction, p=0.0031; Fig. 1D). Because one of our conflict tasks used an appetitive reward, we sought to determine whether 2x IBU impacted task performance by changing hunger. We examined the impact of 2x IBU on chow consumption in the home cage (p=0.90, Fig. 1E) and apple consumption in the Food alone and Food+light tasks (p=0.28 and p=0.60, Fig. 1F–G) and found this dose of IBU did not impact consummatory behavior. The lack of change in consumption of the apple reward in the Food+light task further suggests that 2x IBU did not impact the value of the reward. These collective observations suggest that moderate levels of GHSR activation increases cost-sensitivity during cost-benefit conflict decision-making.

The high-dose 10x IBU did not alter the number of times rats chose to enter the offer zone in any task, including the conflict tasks (p=0.20; Fig. 1H, **fig. S2A-G**), suggesting that GHSR activation impacts cost sensitivity in a dose-dependent manner. However, 10x IBU promoted consummatory behavior (home cage: p=0.0006; Food alone task: p=0.01; Food+light task: p=0.01; **fig. S2H-J**), consistent with other published studies linking GHSR activation and consummatory behaviors (34–36).

### IBU dose-dependently alters activity in a striosome-lateral habenula-dopamine circuit.

We next sought to identify the brain regions impacted by systemic IBU administration. Because GHSRs are widely expressed throughout the brain (37, 38), we quantified IBU-induced expression across fifty brain regions using cFos expression as a proxy for neuronal activity. We examined subdivisions of striatum (**fig. S3A-R;** see Material and Methods Section 5; table S2 for reagents; table S4 for regions) and their inputs and outputs because of the special role that striatum plays in conflict decision-making. We also examined regions that regulate feeding behaviors because GHSR is linked to consummatory behaviors. Several regions exhibited modestly increased cFos expression, but the greatest change, by far, was observed in DMS striosomes (**fig. S3R**); the downstream LHb, which can be either excited or inhibited by striosomal projections (30), had the second largest increase.

**Fig. 2:**
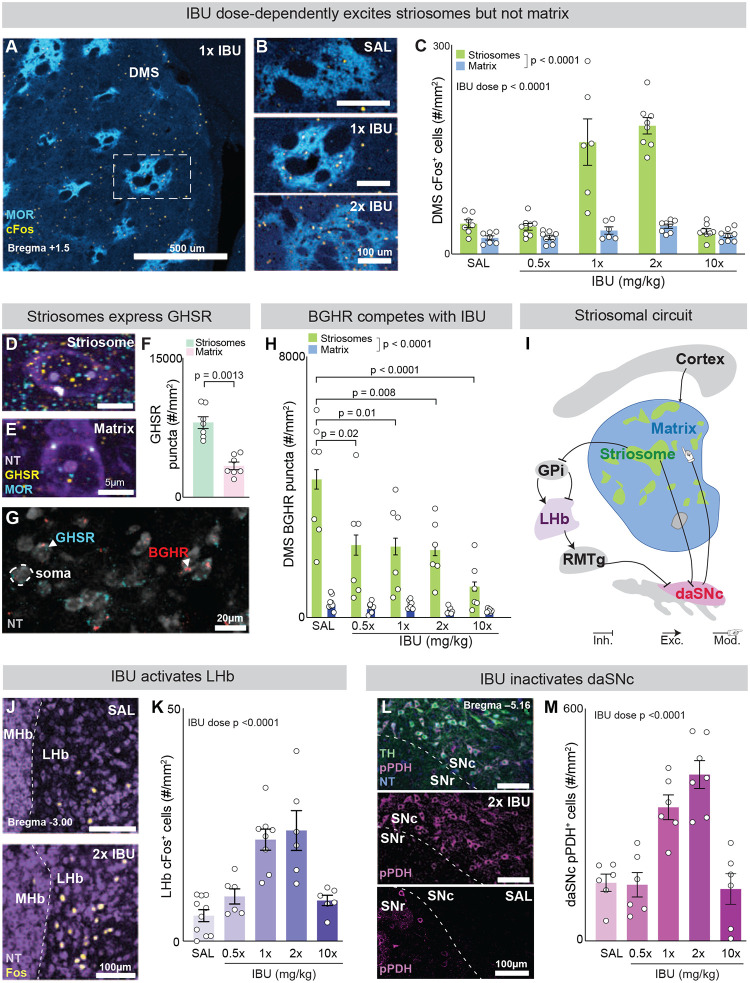
Administration of IBU dose dependently excites striosomes, Lateral Habenula and inactivates dopaminergic neurons of Substantia Nigra pars compacta. **(A-C)** MOR and cFos labeling in DMS striosomes **(A)** following SAL **(B, top)**, 0.5x, 1x **(B, middle)**, 2x **(B, bottom)**, and 10x IBU administration (rats=6–8/group). GHSR modulation dose-dependently increased cFos expression in striosomes **(C**, ANOVA, IBU dose, p<0.0001), but not in matrix (p<0.0001). **(D-H)** GHSR was enriched in **(D)** striosomes relative to **(E)** matrix (**F**, rats=7, t-test, p=0.0013). **(G)** BGHR colocalized with GHSR and selectively labeled striosomes, with significantly greater puncta density in SAL striosomes than SAL matrix (**H**, ANOVA, striosomes vs matrix, p<0.0001). Increasing IBU concentrations progressively reduced BGHR binding across treatment groups (SAL, 0.5x, 1x, 2x, and 10x; rats=6–9/group; ANOVA, effect of IBU dose, p=0.001), consistent with receptor occupancy. **(I)** Schematic of the striosome-LHb-daSNc circuit. Striosomes regulate dopaminergic activity through projections to daSNc and via the GPi-LHb-RMTg pathway. **(J-K)** Intermediate IBU doses (2x) increased LHb cFos expression relative to SAL **(J)**, with a significant effect of IBU concentration across doses (**K**, ANOVA, p<0.0001). **(L-M)** TH/pPDH/NT-stained daSNc sections. Intermediate IBU doses increased pPDH expression relative to SAL **(L)**, indicating reduced dopaminergic activity (**M**, ANOVA, p<0.0001). Mean ± SEM. For additional statistical analyses see table S3.

To determine whether DMS striosomes exhibit dose-dependent changes after GHSR activation, we examined the effect of different doses of IBU (0.5, 1, 2, or 10 mg/kg b.w.; “0.5x” to “10x”) on cFos expression in this pathway. Both 1x and 2x IBU robustly activated DMS striosomes (p<0.0001) without impacting activity in the surrounding matrix (p<0.0001) tissue (Fig. 2A–C; **fig. S3 S-T;** table S3); 10x IBU did not impact cFos expression in either compartment.

Intrigued by the selective increase of striosomal activity at moderate IBU doses, we performed immunostaining for GHSR in the DMS (**fig. S3U-V**), and found strong expression of GHSR in DMS striosomes, but not the surrounding matrix (p=0.0013; Fig. 2D–F). This was surprising because prior publications do not report expression of GHSR in the striatum (37) but these publications did not compare striosomes and matrix. We replicated the striosome-specific expression of GHSR using a biotinylated acyl-ghrelin (BGHR) binding assay (39, 40) (striosome vs matrix, p<0.0001; Fig. 2G–H; **fig S3W**) and confirmed that pre-treatment with IBU dose-dependently reduced the BGHR signal (dose, p=0.001), suggesting that IBU dose-dependently occupies striosomal GHSR.

The striosomes project directly to daSNc (22–28) and indirectly through the GPi-LHb-RMTg circuit (24, 29, 30) (Fig. 2I). Similar to striosomes, we observed similar IBU-induced changes in cFos activity in the LHb (p<0.0001; Fig. 2J–K; **fig. S4A**). Because activation of either the direct or indirect pathways from striosomes to SNc is expected to inhibit dopamine (22, 26, 30, 41, 42), we examined the daSNc for pPDH, a marker for neural inactivity (31). We found that moderate doses of IBU increased inactivity in daSNc (p<0.0001, Fig. 2L–M), which could be explained by the fact that both striosomes and LHb are powerful sources of inhibition of dopamine (22, 26, 41–44). However, following administration of 10x IBU, cFos expression was *increased* in the daSNc (p<0.0001; **fig. S4B-C**), suggesting a dose-dependent activation of dopaminergic neurons by GHSR activation. Interestingly, we found strong correlations between activity of striosomes and LHb, as well as activity of striosomes or LHb and inactivity of daSNc, but not between striosomes and daSNc cFos expression (**fig. S4D-F**). We also confirmed that only 10x IBU increased cFos expression in the Arcuate nucleus of the hypothalamus (ARC), a known site for ghrelin actions (45), which aligns with our behavioral observation that only this dose potentiated food consummatory behaviors (**fig. S4G-H**). Collectively, we found that only moderate levels of GHSR activation activate a striosome-LHb pathway and inhibit downstream daSNc.

### IBU and endogenous acyl-ghrelin dose dependently excite and inhibit striosomes

**Fig. 3:**
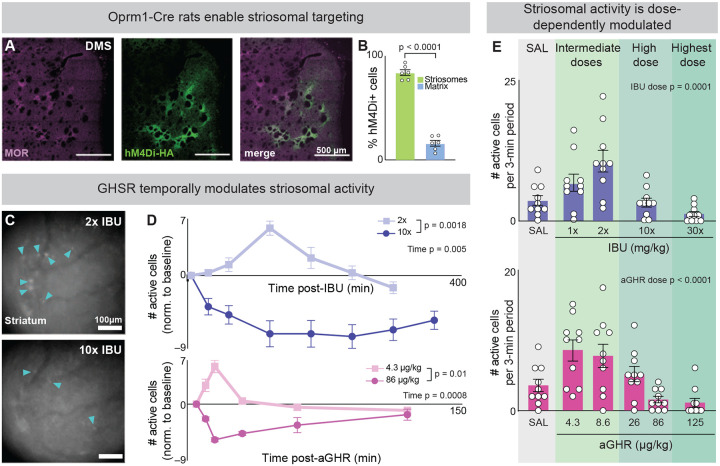
Intermediate doses of IBU and acyl-ghrelin (aGHR) excite, while ultrahigh dose inhibit, striosomes. **(A-B)** Striatal single-channel images of an Oprm1-Cre rat show MOR **(A, left**), DREADD virus (**middle**), and merged signal (**right**). **(B)** Cre-dependent virus expression was predominately localized to MOR-defined striosomes, with minimal expression in matrix (rats=6, t-test, p<0.0001). (**C-E)** In vivo calcium imaging monitored striosomal activity following GHSR activation by IBU and aGHR. **(C)** More active cells were observed following 2x IBU than 10x IBU. **(D)** Time-course analysis of calcium dynamics following IBU and aGHR show low/intermediate-doses (2x IBU and 4.3 μg/kg aGHR) increased striosomal activity, whereas high-doses (10x and 86 μg/kg) decreased activity (IBU, rats=6, p=0.0018; aGHR, rats=5, p=0.01). IBU-induced activity peaked approximately 2 hours after administration and gradually returned toward baseline by 5 hours (time effect, p=0.005), whereas aGHR peaked at 15 min (p=0.0008). **(E)** Striosomal activity across IBU and aGHR concentrations showed a significant effect of dose (IBU, top; aGHR, bottom; rats=8, fields of view=9–10; ANOVA, IBU p=0.0001; aGHR p<0.0001). Both agonists produced an inverted-U response, with intermediate doses increasing and higher doses decreasing the number of active cells. Reduced striosomal activity at higher doses coincided with increased feeding behavior (**fig. S5F-G**). Mean ± SEM. For additional statistical analyses see table S3.

To confirm that GHSR activation produced dose-dependent firing changes in striosomes, we used an Oprm1-Cre rat line (32) to image neuronal activity (Material and Methods section 2; table S2). The *Oprm1* gene encodes the Mu opioid receptor [MOR](46), enabling Cre-dependent gene expression in striosomes. We found the virus is preferential expressed in striosomes (p<0.0001; Fig. 3A–B; table S3). We injected Oprm1-Cre rats with an Adeno-Associated Virus (AAV) expressing floxed GcAMP7f to observe striosome calcium dynamics after IP administration of different doses of IBU or acyl-ghrelin (aGHR) (Fig. 3C; **fig. S5A-C**). Due to the differences in half-lives of IBU and aGHR (~6 hrs vs ~15 min) (33, 47), calcium dynamics were monitored for different amounts of time (5 hrs for IBU; 2 hrs for aGHR; Material and Methods section 6). We calculated the number of active cells during 3 min recordings at different time points (Fig. 3D; **fig. S5D-E**). At low to moderate doses, both IBU and aGHR significantly increased the number of active striosomal cells (IBU p=0.0001; aGHR p<0.0001; Fig. 3E) without impacting feeding, while high doses did not elevate the number of active striosomal cells above baseline (SAL) but did potentiate feeding (**fig. S5F-G**). Surprisingly, an ultra-high dose of IBU (30 mg/kg) or aGHR (125 μg/kg) reduced striosomal activity below baseline levels. Collectively, our data confirm that both IBU and aGHR exhibit an inverted-U relationship between dose and striosomal activity.

### Cost sensitivity in conflict tasks causally depends on striosomes

**Fig. 4:**
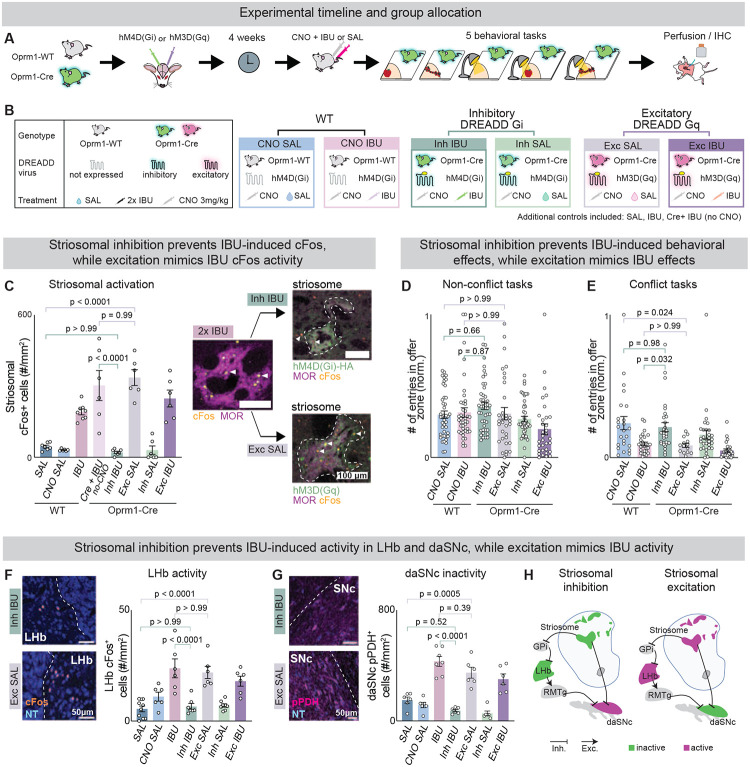
IBU-induced cost avoidance, LHb activity, and daSNc activity causally depend on striosomes. **(A)** Wild-type and Oprm1-Cre rats received Cre-dependent inhibitory hM4D(Gi) or excitatory hM3D(Gq) DREADD targeting striosomes. After 4 weeks of recovery, all rats received CNO and either SAL or 2x IBU before behavioral testing. Following completion of the five-task behavioral battery, rats again received CNO along with SAL or IBU prior to perfusion for immunohistochemical analyses. **(B)** Overview of experimental groups. WT cohorts received CNO+SAL or CNO+IBU. Rats expressing inhibitory DREADD received CNO+SAL (Inh SAL) or CNO+IBU (Inh IBU), whereas rats expressing excitatory DREADD received CNO+SAL (Exc SAL) or CNO+IBU (Exc IBU). **(C-G)** Striosomal inhibition prevented IBU-induced effects, whereas striosomal excitation mimicked the effects of IBU across behavioral testing and the activity markers in striosomes-LHb-daSNc circuit. **(C)** Striosomal cFos expression differed across manipulations (rats=41; ANOVA, p<0.0001; SAL vs Inh IBU, p=0.99; Cre+ IBU vs Exc SAL, p=0.99). **(D)** Striosomal manipulation did not alter non-conflict task performance (rats=54) but significantly affected conflict-task behavior (**E**; p<0.0001). **(F)** LHb cFos and **(G)** daSNc pPDH expression were significantly altered by striosomal manipulation (both p<0.0001). CNO alone produced no detectable effects in striosomes, LHb, or daSNc (all p>0.5). **(H)** Schematic of circuit activity following striosomal manipulation. Mean ± SEM. For additional statistical analyses see table S3.

To determine whether striosomes causally mediate GHSR-induced changes in conflict decision-making and striosome-associated circuit activity, we selectively manipulated striosomal neurons. Oprm1-Cre rats expressing striosomal inhibitory [hM4D(Gi)] or excitatory [hM3D(Gq)] DREADD received clozapine-N-oxide (CNO, 3 mg/kg, IP) together with SAL or 2x IBU before behavioral testing and histological analyses (Fig. 4A–B; for behavioral testing, see Materials and Methods Sections 4.5; for histological analysis see Section 5.6). Wild-type (WT) rats received CNO+SAL or CNO+IBU. We found that striosomal inhibition prevented the IBU-induced increase in cFos expression within DMS striosomes (p<0.0001; Fig. 4C; **fig. S6A-D**; table S3) while increasing pPDH expression (p<0.0001; **fig. S6E-I**). In contrast, striosomal excitation in the absence of IBU (Exc SAL group) increased striosomal cFos levels (SAL vs Exc SAL, p<0.0001).

In the non-conflict tasks, striosomal excitation or inhibition did not alter task performance (Fig. 4D; **fig. S7A-B**). However, in the conflict tasks, striosomal inhibition prevented 2x IBU-induced avoidance of the offer zone (CNO IBU vs Inh IBU p=0.032; Fig. 4E; **fig S7C**). In contrast, striosomal excitation in the absence of IBU reduced entries into the offer zone relative to SAL controls (CNO SAL vs Exc SAL p=0.024), producing behavioral effects similar to 2x IBU. Together, these findings demonstrate that striosomal activity is both necessary and sufficient to heighten behavioral cost sensitivity in conflict decision-making.

We next examined how striosomal manipulations affected activity within the downstream LHb-daSNc circuit. Striosomal inhibition prevented cFos expression in striosomes and LHb and reduced the inactivity marker pPDH in daSNc regardless of SAL- or IBU-treatment (SAL vs Inh IBU: LHb p>0.99, daSNc p=0.52; Fig 4F–G, **fig. S8A-D**). In contrast, striosomal excitation mimicked the effects of 2x IBU throughout the circuit, even in the absence of IBU (IBU vs Exc SAL: LHb p>0.99, daSNc p=0.39; **fig. S8E**). These findings indicate that activity throughout the striosome-LHb-daSNc circuit is determined primarily by striosomal state rather than IBU exposure alone. Control experiments revealed no effect of CNO, DREADD expression, or surgical procedures on circuit activity or striosomal response (**fig. S8F-G**). These findings confirm that the observed behavioral and circuit effects are due to striosomal activity (Fig. 4H).

### Striosomal neurons with IBU-induced hyperactivity project via the LHb pathway

Striosomes send inhibitory projections directly to the daSNc (26, 27) and indirectly through the GPi→LHb pathway (24, 29, 30). The LHb then sends inhibitory projections to the daSNc via the RMTg (43, 44). Thus, activation of either of pathways could suppress dopaminergic output. Because GHSR activation increases striosomal cFos, we next asked whether downstream projection pathways are preferentially activated. To address this, we used projection-specific tracing approaches to compare activity in both circuits: the GPi→LHb pathway and the striosome→daSNc direct pathway, quantifying relative changes in cFos within projection-defined neurons following GHSR activation. To test this, we employed the ‘TRIO’ (Tracing the Relationship between Input and Output) method (48), enabling monosynaptic retrograde labeling of striosomes projecting to LHb via GPi (**fig. S9A-G**; Material and Methods section 7). In a separate cohort of TH-Cre rats we applied retrograde tracing to label striosomes projecting to daSNc neurons (**fig. S9H-K**). We observed similar proportions of RV-mCherry+ striosomal neurons projecting to both the GPi→LHb and daSNc pathways (p=0.76; **fig. S9L-N**). However, when comparing IBU-induced cFos, we found a significantly greater proportion of RV-mCherry+ striosomal neurons colocalized with cFos in the GPi→LHb pathway compared to the daSNc pathway (p=0.033). These results indicate that IBU-induced striosomal activity preferentially engages the GPi→LHb circuit, suggesting that daSNc inactivity is more likely driven by striosomal projections to the LHb. Consistent with this, selective striosomal inhibition reduced LHb activity to baseline levels and decreased inactivity of daSNc (Fig. 4F–G), linking this pathway to functional circuit output.

### A decision-space model of the striosomal circuit

**Fig. 5:**
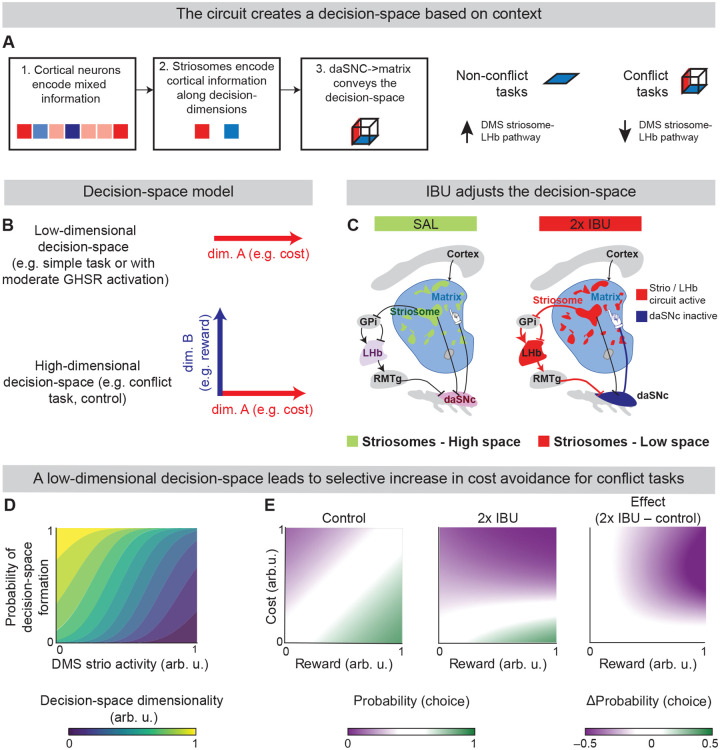
Computational modeling of striosomes and GHSR-activation interactions during decision-making. **(A)** Circuit model illustrating how striosomes encode cortical information along orthogonal decision-dimensions that are integrated by daSNC-matrix pathways to construct a “decisionspace”. Task conflict determines the number of recruited dimensions. The model demonstrates that striosomes are more active in low conflict/1D tasks and less active in conflict/high dimensional tasks (18, 19, 21). See note S1 for model details. **(B)** Decision-space dimensionality is jointly shaped by task demands and IBU dose. **(C)** At 2x IBU, elevated striosome→LHb activity inhibits downstream dopaminergic signaling, compressing the decision-space. **(D)** Simulation of the theoretical relationship between DMS striosomal activity and decision-space dimensionality. For simplicity, only reward and cost dimensions are shown, though additional dimensions (novelty, effort, etc.) may contribute. **(E)** Model demonstrates approach-avoid behavior across reward-cost space. Purple denotes avoidance while green indicates approach. Elevated striosome→LHb activity selectively increases avoidance in high-reward/high-cost regions, consistent with observed light-avoidance behavior, while minimally affecting low-conflict choices. At baseline **(left)** striosomes rule with unaltered context gating creating a balance between reward and cost. During the 2x IBU condition **(middle)**, decision-making boundaries are shifted towards avoidance because of striosome→LHb hyperactivity. The difference (**right**, baseline minus IBU) shows this increase in avoidance.

To explain how GHSR activity alters behavior, we integrated our dose-dependent findings on behavior and on striosomal circuit activity into our “decision-space” framework (19) (see Note S1, table S5, Material and Methods section 8). In this model, the cortex broadcasts multiple streams of information (e.g. reward value of a toy or food, aversiveness of bright light, reward novelty, motor costs, internal hunger and fear signals) to the striatum (Fig. 5A). Each stream is represented as a distinct “decision-dimension” in striosomal subpopulations (Fig. 5B). The overall excitability of the striosomal network inversely determines dopamine release into the matrix (19, 49). When striosome activity is lower (e.g., under SAL), many dimensions signaled by dopamine pass through into the downstream matrix, forming a broad, high-dimensional decision-space. When striosome activity is higher, less dopamine is released into the matrix, reflecting the transmission of fewer dimensions. In our conflict tests (Food+light), two key dimensions must normally be weighed: the food reward and the aversive light. Theoretically, certain doses of IBU induce increased activity in the striosomes and LHb, narrowing the decision-space to overemphasize cost dimensions, driving avoidance without affecting feeding (Fig. 5C). This could explain why our empirical results hold specifically for conflict: the narrowing of information only changes behavior when there are multiple competing signals to filter (Fig. 5D–E).

We further developed a conceptual model to explain why these behavioral effects follow an inverted-U curve. This model suggests that striosomal engagement is strongly dependent on GHSR activation. At low doses, striosomal activity is unchanged, leaving behavior unaffected. At moderate doses, striosome activity rises, narrowing the decision-space to enable cost-sensitive choices during conflict. However, at high doses, striosomal activity returns to baseline or is inhibited (**fig. S10A-B**). This conceptual model predicts that the relationship between GHSR activity and task performance is not fixed but must be calibrated to the specific complexity and demands of the task at hand to ensure the correct information is prioritized (**fig. S10C**).

## Discussion

Our results demonstrate a novel role for acute GHSR activation in striosomal circuit dynamics and striosome-dependent conflict decision-making. Our results are surprising for two reasons. First, it is commonly thought that striatum lacks GHSR expression. Robust striosomal GHSR expression may have been overlooked because striosomes only account for approximately 10–20% of total striatal volume (20, 46); when GHSR expression is computed across the whole striatum, the low expression in the surrounding matrix will dominate the average expression calculated. Second, despite well-characterized roles in aversion and reward, we showed here that moderate activation of GHSR dramatically reshapes conflict decision-making (Fig. 1).

Given the ubiquity of GHSR throughout the brain (37, 38), it is reasonable to question whether our behavioral and circuit effects can be attributed to activation of GHSR within the striosomes *per se*. Several pieces of evidence support this claim. First, we demonstrate that selective chemogenetic inhibition of striosomes prevented IBU-induced changes in gene expression throughout the downstream striosomal circuit, as well as IBU-induced changes in conflict behavior (Fig. 4). This shows that the striosomes are necessary for these IBU-induced changes. Second, the striosomes are known to play a special role in reward-cost conflict decision-making (18, 19) and do not regulate single valence choices (Fig. 1,4). If our results were attributable simply to actions of GHSR on reward valuation, for example, then one would expect to see alterations in both conflict decision-making and single valence decisions.

Our results draw attention to three important points about the role of GHSRs in valenced behaviors. We demonstrated a similar impact of GHSR activation on conflict decision-making when either food or non-food rewards were used (Fig. 1C–D). This agrees with a relatively small number of studies showing that ghrelin, a gut-derived hormone, has a broader impact on reward-related processing than simply through modulating appetite (9–13) (see note S1, table S5). Reinforcing this concept, we also observed that activation of hypothalamic regions that drive hunger (**fig. S4G**), as well as increased consummatory behaviors (**fig. S2H-J**, **S5F-G**) presumably reflecting increased hunger, only occurred with high doses of IBU that did not impact conflict choice behavior. This suggests that hunger circuits are relatively insensitive to changes in GHSR signaling compared to striatal circuits that regulate conflict decision-making and that there may be a “trade-off” at high levels of GHSR signaling where food-seeking is prioritized over more complex decision-making. This may be consistent with the observation that high levels of endogenous circulating ghrelin are associated with hunger: ghrelin doubles across the 24-hour day in ad libitum-fed mice (50), as well as in anticipation of meals in both rodents and humans (51, 52). Lastly, our findings provide a powerful demonstration of the importance of dose-response curves when manipulating GHSR (Fig. 2, 3E). We demonstrate that changes in the striosome-GPi-LHb-daSNc circuit occur only at moderate levels of GHSR activation, with little no activity observed at low or high doses. Pharmacological studies examining the role of GHSRs in valenced behavior have sometimes produced conflicting results [for example, in anxiety; reviewed in (53)], and this may, in part, be because only single doses of GHSR agonists or antagonists were tested.

Previous studies showed that the striosomes regulate dopamine release by the SNc either directly or through an LHb (22, 26, 30), but it is unknown what conditions preferentially activate one pathway over the other. Here we show that moderate GHSR activation is an important switch for activating the LHb pathway and reducing neuronal activity in the daSNc (Fig. 4, **fig. S9**). The mechanism for this preferential switch is not understood. One possibility is that GHSR is preferentially expressed by striosomal neurons projecting to the LHb pathway. Regardless of the mechanism, our study extends the striosomal literature by demonstrating an important activator of the LHb pathway.

An important remaining question is why high versus moderate doses of ghrelin receptor agonists have differential effects on striosomal activity. GHSRs can form heterodimers with multiple other receptors (54, 55) known to be abundant in striosomal neurons, including dopamine 1 receptors, dopamine 2 receptors (56, 57), serotonin 2C receptors (58), and cannabinoid type 1 receptors (59). The affinity of ghrelin agonists for GHSR may be different across these heterodimers, and thus escalating GHSR agonism may recruit new intracellular signaling pathways as higher affinity heterodimers are fully occupied. Addressing this possibility is an important and challenging direction for future studies.

In addition to conflict decision-making behavior, we found that GHSR activation impacted nuanced aspects of motor behavior during the tasks (**fig. S11–13**). These changes could reflect the role of striatum in control of movement (60–62) or downstream inhibition/reduction of daSNc activity (see note S2). One distinct motoric feature of GHSR stimulation with moderate doses or chemogenetic excitation of striosomes is increased head-body rotation; this could be a form of information gathering, and may be similar to the increased sniffing (63) and decreased movement (41) reported in another study examining increased striosome activity and movement. This effect also might be due to GHSR activation at other brain sites. For example, GHSR activation promotes olfactory sampling (64).

The striatum can be subdivided in multiple ways, including across lateral and medial dimensions (65, 66). Though we focused here on DMS striosomes because of their role in conflict decision-making (18), we did observe similar binding of BGHR expression levels in the striosomes of the dorsolateral striatum (DLS) (**fig. S14A-B**), which encodes values (67) of habitual and familiar movements (68). Following 2x IBU, we found that DLS striosomes expressed more of the inactivity marker pPDH, and less cFos than DMS (**fig. S14C-H**), suggesting that moderate GHSR activation could promote a shift away from habitual to more exploratory behaviors. The difference in DLS and DMS responses to GHSR agonist administration could be driven by the difference in the proportion or distribution of direct and indirect pathway neurons across medial-lateral striatal gradients (24, 41, 56, 57). The difference may also be attributed to the interaction of dopamine and GHSR receptors (40, 69) when direct and indirect pathway neurons have different proportions across striatal gradients. This might explain the pattern of DMS striosome hyperactivity and DLS striosome hypoactivity observed in cocaine exposure (70) and stress (71), two conditions associated with elevations of endogenous ghrelin (72, 73). Our modeling work predicts that this dual GHSR-mediated shift across the DLS and DMS could allow for intricate control over information processing, where complementary “decision-spaces” allow parallel processing of competing behavioral strategies (**fig. S14I-J**).

Our findings have sweeping implications: elevated ghrelin levels, aberrant striosomal activity, and impaired decision-making are observed in chronic stress (21, 73), PTSD (16), substance use disorders (17, 70, 74), and suicide (15, 75). Our study identifies an intriguing new circuit mechanism that could potentially link disorder-related changes in endogenous ghrelin to changes in striosomal activity and aberrant cost-benefit decision-making and suggests that both GHSR antagonists and high levels of GHSR agonists could reduce striosomal hyperactivity and promote healthy cost-benefit decision-making. Our computational model defines the effect of GHSR stimulation on decision-making and paves the way for future work that could provide context-dependent calibration to optimize decision-making (**fig. S15**; note S3; table S6). Future research should explore whether chronic elevation of ghrelin activates the circuit described here or whether there are additional adaptations that should be considered in the context of therapeutic interventions for psychiatric disorders in which endogenous ghrelin is elevated. This project has some limitations (note S4) but despite these limitations, our research demonstrates that GHSR stimulation via IBU has separable behavioral functions, from altering conflict decision-making to appetitive pursuit. We also provide evidence for GHSR expression in the striosomes and demonstrate that striosomal activity is a necessary and causal component of IBU-induced changes to conflict decision-making.

## Supplementary Material

Supplementary Files

This is a list of supplementary files associated with this preprint. Click to download.

- SupplementaryMaterialsXMethodsfinalNaturecompressed3.pdf

## Data Availability

All raw, analyzed data, and modeling codes have been deposited in publicly accessible repositories, with complete information and DOIs listed in the Supplementary Materials table S7.
